# Effectiveness of lifestyle change plus dental care (LCDC) program on improving glycemic and periodontal status in the elderly with type 2 diabetes

**DOI:** 10.1186/1472-6831-14-72

**Published:** 2014-06-16

**Authors:** Saruta Saengtipbovorn, Surasak Taneepanichskul

**Affiliations:** 1 Health center 54, Bangkok, Thailand; 2 College of Public Health Sciences, Chulalongkorn University, Bangkok, Thailand

**Keywords:** Lifestyle change, Dental care, Glycemic status, Periodontal status, Elderly, Type 2 diabetes

## Abstract

**Background:**

Currently, there is an increased prevalence of diabetes mellitus among the elderly. To minimize adverse effects on glycemic control, prevention and management of general and oral complications in diabetic patients is essential. The purpose of the present study is to assess the effectiveness of a Lifestyle Change plus Dental Care (LCDC) program to improve glycemic and periodontal status in the elderly with type 2 diabetes.

**Methods:**

A quasi-experimental study was conducted in Health Centers 54 (intervention) and 59 (control) from October 2013 to January 2014. 66 diabetic patients per health center were included. At baseline, the intervention group attended a 20 minute lifestyle and oral health education program, individual lifestyle counseling using motivational interviewing (MI), application of self regulation manual, and individual oral hygiene instruction. The intervention group received booster education every visit by viewing a 15 minute educational video. The control group received a routine program. Participants were assessed at baseline and 3 month follow up for glycosylated hemoglobin (HbA1c), fasting plasma glucose (FPG), body mass index (BMI), periodontal status, knowledge, attitude and practice of oral health and diabetes mellitus. Data were analyzed by using descriptive statistic, Chi-square test, Fisher’s exact test, t-test, and multiple linear regression.

**Results:**

After the 3 month follow up, a multiple linear regression analysis showed that the intervention group was significantly negatively correlated in both glycemic and periodontal status. Participants in the intervention group had significantly lower glycosylated hemoglobin (HbA1c), fasting plasma glucose (FPG), plaque index score, gingival index score, pocket depth, clinical attachment level (CAL), and percentage of bleeding on probing (BOP) when compared to the control group.

**Conclusions:**

The combination of lifestyle change and dental care in one program improved both glycemic and periodontal status in the elderly with type 2 diabetes.

**Trial registration:**

ClinicalTrials.in.th: TCTR20140602001.

## Background

The elderly are at high risk of chronic conditions including diabetes mellitus (DM), arthritis, congestive heart failure, and dementia [1]. Furthermore, the prevalence of DM and oral manifestations of DM including periodontal disease also increase in the elderly [2].

Currently, many countries face the problem of increased prevalence of DM, which is a chronic, systemic metabolic disorder. DM causes morbidity and mortality due to long-term complications, which affect the important organs [2]. Clinical complications of DM include retinopathy, nephropathy, neuropathy, macro-vascular disease, delayed wound healing, and periodontal disease [3]. Patients with type 2 diabetes have to make multiple choices about the management of their condition, such as, appropriate dietary intake, physical activity, and adherence to drugs [4].

Periodontal disease, an inflammatory disease affecting the periodontium, is a complication of type 2 diabetes associated with health outcomes due to systemic inflammation [2]. Periodontal disease and DM have a bidirectional relationship [2]. The effects of DM on periodontal health and periodontal infection also affect glycemic control. Furthermore, periodontal infection increases the risk for developing DM complications [2]. It is important to minimize the adverse effects of oral complications on glycemic control in diabetic patients, particularly periodontal disease, through prevention and management [2].

There have been many studies of diabetes intervention programs to prevent and control diabetic complications by decreasing HbA1c including using nurse practitioners for lifestyle intervention in Dutch primary care [5], using delivery meals and dietary counseling to reduce body weight in Japan [6], using lifestyle change programs (dietary counseling and exercise) to reduce the proportion of metabolic syndrome [7] and using diabetes empowerment to increase medication adherence and self-care behavior [8]. Furthermore, there have been periodontal intervention programs in others countries which have emphasized periodontal therapy including tooth brushing instruction, oral health education, and supra-gingival scaling. All of these programs reportedly improved glycemic control by decreasing HbA1c and periodontal status with statistically significant differences [9-11]. However, Promsudthi et al. [12], who carried out their study in Thailand, found a decrease of HbA1c 3 months after periodontal treatment of scaling and root planing without a statistically significant difference [12]. Although, diabetes intervention programs can prevent and control diabetic complications by decreasing HbA1c and periodontal intervention programs also decreased HbA1c and periodontal status, decreasing HbA1c from lifestyle change alone may not prevent periodontal disease; it only decreases the risk of periodontal infection [2]. The most important method for treating periodontal disease is intensive oral hygiene instructions including teaching tooth brushing, flossing, and the use of others devices [13]. Periodontal treatment programs improved periodontal status [9-12]. To control DM, patients should maintain healthy lifestyles and routinely control health levels, healthy eating habits, regular physical activity, and take diabetes medicine [14]. Combined lifestyle change and periodontal care intervention are needed to prevent dental complication.

The objective of the present study was to assess the effectiveness of the Lifestyle Change plus Dental Care (LCDC) program to improve glycemic and periodontal status in the elderly with type 2 diabetes.

## Methods

The quasi-experimental study was conducted in two health centers located in Bangkok, Thailand from October 2013 to January 2014. Health Centers 54 and 59 were selected from 68 health centers in Bangkok because these health centers serve a population with similar socio-demographic characteristics, have scheduled appointments and have at least 500 patients in their Diabetes Clinics. The two health centers were randomly assigned to intervention (Health Center 54) and control (Health Center 59) groups. Systematic sampling was used to select 66 participants in each health center. The sample size was calculated based on a previous study [12]. The average HbA1c level of the intervention and the control groups were 8.78% and 9.28%, respectively and the pooled variance was 0.88 [12]. The sample size required in each group in the current study was 55 with 80% power at the 5% significant level. 20% was increased for refusal and attrition in each group so the total sample size in each group was 66 and overall sample size was 132 participants.

The inclusion criteria for both male and female participants included patient age over 60 years with type 2 diabetes and at least 16 natural teeth (acceptable for scoring plaque and gingival index). The patients who had serious systemic diseases or complications, blood disease, liver damage, kidney disease, severe chronic periodontitis, communicable disorder, could not speak Thai language, or did not agree to participate, were excluded.

### Training of interviewers

The interviewers were standardized by attending a 2–day training program. Two nurse practitioners were trained in Motivational Interviewing (MI) for lifestyle change and dental care including dietary counseling, physical activity, quiting smoking, and oral health care, by experts in this field. Moreover, the same two nurse practitioners and two dental assistants attended a one day training program for the education and teaching technique, by experts in education, diabetes, and oral health.

### Intervention

Lifestyle Change plus Dental Care (LCDC) program is based on the health belief model, social cognitive theory, and cognitive-behavioral theory [15]. At baseline, participants received a 20 minute lifestyle and oral health education program, by trained nurse practitioners, which emphasized type 2 diabetes complications, the prevention of general and oral health complications, the relationship between type 2 diabetes and oral complications, and oral health care. Then participants received individual lifestyle counseling by MI, application of self regulation manual and selected their goal of lifestyle and oral health care change with trained nurse practitioners. The content of the lifestyle counseling and self regulation manual were consistent with lifestyle change and oral health education. The goals included loss of body weight, eating healthy food (fruits and vegetables), eating more high-fiber foods, eating less sugar, exercising for more than 30 minutes at least 3–5 times/week, quiting smoking, tooth brushing after meals, and using dental floss at least 1 time/day. Individual oral hygiene instruction by trained dental assistants was also conducted in the dental room. The content included tooth brushing with fluoride toothpaste, using dental floss or other devices such as inter-proximal brush, cleaning dentures, and how to check oral health by themselves.

In the 1st and 2nd month, participants received an educational booster by viewing a 15 minute educational video covering all of the abovementioned points. Furthermore, the goal of lifestyle and oral health care change was boosted by nurse practitioners.

A focus group discussion was used to develop a slide presentation for lifestyle change and oral health education, self regulation manual, and the 15 minute educational video by brainstorming ideas from doctors, nurse practitioners, dentists, dental assistants, and a representative of diabetic patients. The slide presentation, self regulation manual, and educational video were validated by an expert in education, an expert in diabetes and an expert in dentistry. A pretest of the three items was also conducted.

### Control group

The routine program in the diabetes clinic, which the participants in the control group attended, included seeing a doctor, measuring fasting plasma glucose (FPG), collecting diabetic medicine from a pharmacist, and making an appointment for their next visit.

### Outcome measures

Participants in both groups received oral examination, blood sample testing, and face-to-face interview at baseline and 3 month follow up. The single-blind technique was used. The participants did not know if they were in the intervention or the control group.

Oral examination was done by two calibrated dentists. Inter-examiner reliability was tested by using Cronbach’s Coefficient Alpha. Another 5 diabetic patients were examined for periodontal status (plaque index, gingival index, pocket depth, gingival margin, and percentage of bleeding on probing (BOP)) by three dentists included one expert in periodontics (gold standard) and the other two dentists who conducted the present study to measure the agreement between examiners. The Cronbach’s Coefficient Alpha was 0.85. Plaque index score, gingival index score, pocket depth, clinical attachment level (CAL), and percentage of bleeding on probing (BOP) [16] were used to find periodontal status. Pocket depth was measured by a level from the gingival margin to the most coronal extension of the epithelial attachment [17]. Gingival recession was measured by a level from cemento-enamel junction to the gingival margin [17]. The pocket depth and gingival recession of the six surfaces (mesiobuccal, midbuccal, distobuccal, mesiolingual, midlingual, and distobuccal) of every tooth in the diabetic patient’s mouth were recorded by using a periodontal probe [18]. Clinical attachment level (CAL) was calculated by measuring pocket depth plus gingival recession [18].

Blood sample testing was done by nurse practitioners. Biomedical outcomes included glycosylated hemoglobin (HbA1c), fasting plasma glucose (FPG), and body mass index (BMI).

The questionnaire was validated by three experts in public health. The Item-Objective Congruence Index (IOC) was 0.83. A pilot study was carried out to test the reliability of the questionnaire. The Cronbach’s Coefficient Alpha was divided into 4 parts; knowledge toward oral health and DM: 0.84, attitude toward oral health and DM: 0.87, oral health behaviors: 0.77, and practice toward DM: 0.89. The questionnaire included general characteristics, BMI, knowledge and attitude toward oral health and DM, oral health behaviors, and practice toward DM.

### Statistical analysis

Descriptive statistic, Chi-square test, Fisher’s exact test, and t-test were used to compare the difference between the intervention and the control groups at baseline. T-test was used to compare the difference of biomedical outcomes, periodontal status, knowledge and attitude toward oral health and DM at baseline and 3 months follow up between the intervention and the control groups. Chi-square test and Fisher’s exact test were used to explore the effect of the intervention on oral health behaviors and practice toward DM. Multiple linear regression was also used to measure the relationship. The Enter Method was used to include variables in the regression models. Group affiliation, age, gender, smoking, BMI, and the respective baseline measures were adjusted for glycemic status (FPG and HbA1c). In regard to periodontal status (plaque index, gingival index, pocket depth, CAL, and BOP), group affiliation, age, gender, smoking, and the respective baseline measures were adjusted. Data were analyzed by SPSS statistical package version 16.0. All analysis used a 95% confidence interval (CI), and statistically significant p-value of less than 0.05.

### Ethical consideration

Ethics approval was granted from the Ethics Review Committee for Research Involving Human Research Subjects, Health Science Group, Chulalongkorn University (No. 123.1/56). Informed consent was signed by all participants.

## Results

### Study population

Of the 132 participants who enrolled at baseline (66 intervention and 66 control), 130 (98.5%) were eligible for follow up at 3 months. Of those who were not eligible, 1 participant in the intervention group was too ill to follow up (paralysis) and 1 participant in the control group moved to live in another province.

### Baseline characteristics

Among the 132 diabetic patients (66 diabetic patients per group), most of the participants in each group were female. The average age of the intervention and the control groups were 63.83 (4.51) years and 64.06 (5.53) years, respectively. There were no statistically significant differences of gender, age, educational level, BMI, health insurance, duration of having diabetes, and smoking between the intervention and the control groups (p = 0.856, 0.357, 0.790, 0.057, 0.643, 0.118 and 0.474, respectively) (The details are demonstrated in Table 1).

**Table 1:** Baseline characteristics (N = 132)

**Variables**	**Intervention group**	**Control group**	p-value*
	**(n = 66) (%)**	**(n = 66) (%)**	
**Gender**			
Male	23 (34.8)	24 (36.4)	0.856
Female	43 (65.2)	42 (63.6)	
**Age**			
60–69 years	56 (84.8)	55 (83.3)	0.357
70–79 years	10 (15.2)	9 (13.6)	
≥80 years	0 (0.0)	2 (3.1)	
**Educational level**			
Illiteracy	2 (3.0)	3 (4.5)	0.790
Primary school	53 (80.3)	48 (72.8)	
Secondary school	8 (12.2)	9 (13.7)	
Vocational school	2 (3.0)	3 (4.5)	
Bachelor degree	1 (1.5)	3 (4.5)	
**BMI (kg/m**^**2**^**)**			
Mean (S.D.)	25.30 (3.57)	26.63 (4.37)	0.057
Min-max	17.95–34.60	18.93–36.50	
**Health insurance**			
Universal coverage	55 (83.4)	59 (89.4)	0.643
Universal coverage (other)	3 (4.5)	2 (3.0)	
Government/state enterprise officer	7 (10.6)	5 (7.6)	
No	1 (1.5)	0 (0.0)	
**Duration of being diabetes (years)**			
Mean (S.D.)	6.86 (5.16)	8.42 (6.19)	0.118
Min-max	1–20	1–25	
**Smoking**			
Never	57 (86.4)	58 (87.8)	0.474
Ever	7 (10.6)	4 (6.1)	
Current smoker	2 (3.0)	4 (6.1)	

p by chi-square test or t-test.

*Statistical significances of difference: p < 0.05.

### Biomedical outcomes

Among the remaining 130 participants (65 intervention and 65 control), HbA1c in the intervention group decreased from 7.39 (1.19) % at baseline to 7.10 (1.04) % at 3 month follow up. Whereas, in the control group, HbA1c increased from 7.68 (1.48) % at baseline to 7.77 (1.46) % at 3 month follow up. FPG in the intervention group decreased from 143.83 (38.78) mmol/l at baseline to 129.57 (21.24) mmol/l at 3 month follow up. Whereas, in the control group, FPG increased from 152.91 (51.35) mmol/l at baseline to 158.32 (47.28) mmol/l at 3 month follow up. There were statistically significant differences of both HbA1c and FPG between the intervention and the control groups at 3 month follow up (p = 0.003 and <0.001, respectively). However, there was no statistically significant difference in BMI between the intervention and the control groups at 3 month follow up (p = 0.057) (Table 2).

**Table 2:** T-test for biomedical outcomes, periodontal status, knowledge and attitude toward oral health and DM (N = 130)

**Variables**	**Intervention group**		**Control group**		p-value*
	**n = 65**		**n = 65**		
	**Mean**	**Standard deviation**	**Mean**	**Standard deviation**	
**HbA1c**					
Baseline	7.39	1.19	7.68	1.48	0.217
3 months	7.10	1.04	7.77	1.46	0.003
**FPG**					
Baseline	143.83	38.78	152.91	51.35	0.258
3 months	129.57	21.24	158.32	47.28	<0.001
**BMI**					
Baseline	25.35	3.57	26.66	4.40	0.064
3 months	25.57	3.64	26.88	4.11	0.057
**Plaque index score**					
Baseline	0.59	0.42	0.63	0.41	0.544
3 months	0.26	0.31	0.45	0.45	0.006
**Gingival index score**					
Baseline	0.64	0.41	0.77	0.48	0.139
3 months	0.27	0.29	0.48	0.40	0.001
**Pocket depth**					
Baseline	2.35	0.55	2.39	0.81	0.719
3 months	2.04	0.47	2.54	0.88	<0.001
**CAL**					
Baseline	3.33	0.86	3.68	1.31	0.079
3 months	2.96	0.85	3.64	1.37	0.001
**BOP**					
Baseline	35.36	25.83	37.32	22.17	0.644
3 months	17.66	22.18	38.22	33.36	<0.001
**Overall knowledge**					
Baseline	7.19	2.36	7.04	2.27	0.705
3 months	9.48	0.70	7.29	2.23	<0.001
**Oral knowledge**					
Baseline	3.68	1.73	3.51	1.60	0.564
3 months	4.77	0.52	3.63	1.36	<0.001
**DM knowledge**					
Baseline	3.53	1.09	3.58	0.97	0.799
3 months	4.70	0.28	3.66	1.17	<0.001
**Overall attitude**					
Baseline	43.75	4.42	41.80	6.57	0.057
3 months	47.82	3.41	41.44	5.27	<0.001
**Oral attitude**					
Baseline	21.54	2.95	20.78	3.15	0.161
3 months	23.85	1.80	20.58	3.18	<0.001
**DM attitude**					
Baseline	22.25	2.32	21.74	2.57	0.239
3 months	23.92	1.81	20.86	2.62	<0.001

p by t-test.

*Statistical significances of difference: p < 0.05.

### Periodontal status

Among the 130 participants (65 intervention and 65 control), the plaque index score and gingival index score decreased from baseline to 3 month follow up in both the intervention and the control groups with statistically significant differences between the intervention and the control groups at 3 month follow up (p = 0.006 and 0.001, respectively). Pocket depth and CAL decreased from baseline to 3 month follow up only in the intervention group. Whereas, in the control group, pocket depth increased and CAL was equal at baseline and 3 month follow up. The percentage of BOP decreased from baseline to 3 month follow up in the intervention group. Whereas, in the control group, the percentage of BOP increased from baseline to 3 month follow up. There were statistically significant differences between the intervention and the control groups at 3 month follow up of pocket depth, CAL, and BOP (p < 0.001, 0.001 and, <0.001, respectively) (Table 2).

### Knowledge and attitude toward oral health and DM

The average score of overall knowledge, oral health knowledge, and diabetes knowledge increased from baseline to 3 month follow up only in the intervention group with statistically significant differences between the intervention and the control groups at 3 month follow up (p < 0.001, <0.001 and <0.001, respectively) (Table 2).

The average score of overall attitude, oral health attitude, and diabetes attitude increased from baseline to 3 month follow up only in the intervention group with statistically significant differences between the intervention and the control groups at 3 month follow up (p < 0.001, <0.001 and <0.001, respectively) (Table 2).

### Oral health behaviors

Every participant (100%) in both the intervention and the control groups reported regular cleaning of their oral cavity by tooth brushing at baseline and 3 month follow up (Table 3).

**Table 3:** Chi-square test and Fisher-exact test for oral health behaviors and practice toward DM, at 3 month follow up (N = 130)

**Variables**	**Intervention group (n = 65)**	**Control group (n = 65)**	p-value*
	**no. (%)**	**no. (%)**	
**Oral health behaviors**			
**Tooth brushing** (yes)	65 (100.0)	65 (100.0)	-
**Mouth rinse** (yes)	32 (49.2)	24 (36.9)	0.157
**Salt solution** (yes)	32 (49.2)	19 (29.2)	0.020
**Dental floss** (yes)	46 (70.8)	5 (7.7)	<0.001
**Tooth pick** (yes)	33 (50.8)	40 (61.5)	0.216
**Inter-proximal brush** (yes)	23 (35.4)	20 (30.8)	0.576
**Have had dental treatment, previous 3 months** (yes)	24 (36.9)	15 (23.1)	0.085
**Practice toward DM** **Exercise**			
> 5times/week	19 (29.2)	6 (9.2)	< 0.001
2-5 times/week	24 (36.9)	22 (33.8)	
1 time/week	12 (18.5)	9 (13.8)	
2-3 times/month	7 (10.8)	5 (7.7)	
Never	3 (4.6)	23 (35.5)	
**Tested weight**			
> 1 time/month	22 (33.8)	13 (20.0)	0.075
1 time/month	43 (66.2)	52 (80.0)	
**Diet modification** (yes)	61 (93.8)	46 (70.8)	0.001
**Forgot to take any drugs prescribed** (yes)	28 (43.1)	30 (46.2)	0.724
**Eye examination, last year** (yes)	56 (86.2)	50 (76.9)	0.175
**Foot examination, last year** (yes)	57 (87.7)	16 (24.6)	< 0.001
**Always wear covered shoes** (yes)	41 (63.1)	19 (29.2)	< 0.001
**Screening of feet**			
Everyday	50 (76.9)	39 (60.0)	0.044
Sometimes	12 (18.5)	15 (23.1)	
Rarely/never	3 (4.6)	11 (16.9)	

p by chi-square test or Fisher-exact test.

*Statistical significances of difference: p < 0.05.

At baseline, there were no statistically significant differences in the use of mouth rinse, salt solution, dental floss, toothpick, inter-proximal brush, and having had dental treatment between the intervention and the control groups (p = 0.856, 0.291, 0.804, 0.722, 0.176, and 0.148, respectively).

After 3 month follow up, the participants in the intervention group were more likely to use mouth rinse, salt solution, dental floss, and inter-proximal brush. However, only salt solution and dental floss had statistically significant differences (p = 0.020 and < 0.001, respectively). The participants in the intervention group were less likely to use toothpicks with no statistically significant difference (p = 0.216). Furthermore, the participants in the intervention group were more likely to have had dental treatment in the previous 3 months with no statistically significant difference (p = 0.085) (Table 3).

### Practice toward DM

At baseline, there were no statistically significant differences in exercise, tested weight, diet modification, forgetting to take any prescribed drugs, eye examination, foot examination, always wearing covered shoes, and screening of feet between the intervention and the control groups (p = 0.310, 0.397, 0.518, .0384, 0.394, 0.170, 0.282, and 0.337, respectively).

After 3 month follow up, the percentage of participants who exercised more than 5 times/week in the intervention group (29.2%) was more than the control group (9.2%). Moreover, the participants who never exercise in the intervention group (4.6%) was less than the control group (35.5%). Exercise had a statistically significant difference between the intervention and the control groups (p < 0.001). The percentage of participants in the intervention group (93.8%) who modified diet was higher than the control group (70.8%) with a statistically significant difference (p = 0.001). The percentage of participants in the intervention group who received foot examination, always wore covered shoes, and participated in screening of feet were higher than the control group with statistically significant differences (p <0.001, <0.001, 0.044) (Table 3).

### Multiple linear regression analysis

In the multiple linear regression analysis, the intervention group was significantly negatively correlated in both glycemic and periodontal status at 3 month follow up. FPG at 3 month follow up was significantly correlated with group affiliation and FPG at baseline (*R*^*2*^ = 0.539, *p* <0.001). HbA1c at 3 month follow up was significantly correlated with group affiliation, smoking, and HbA1c at baseline (*R*^*2*^ = 0.757, *p* <0.001) (Table 4). The plaque index score at 3 month follow up was significantly correlated with group affiliation, smoking, and the plaque index score at baseline (*R*^*2*^ = 0.238, *p* <0.001). The gingival index score at 3 month follow up was significantly correlated with group affiliation and the gingival index score at baseline (*R*^*2*^ = 0.200, *p* <0.001). Pocket depth at 3 month follow up was significantly correlated with group affiliation and pocket depth at baseline (*R*^*2*^ = 0.533, *p* <0.001). CAL at 3 month follow up was significantly correlated with group affiliation and CAL at baseline (*R*^*2*^ = 0.721, *p* <0.001). The percentage of BOP at 3 month follow up was significantly correlated with group affiliation and BOP at baseline (*R*^*2*^ = 0.318, *p* <0.001) (Table 5).

**Table 4:** Multiple linear regression analysis for glycemic status (FPG and HbA1c) (N = 130)

**Variables**	**FPG (3rd month)**	**HbA1c (3rd month)**
	Parameter estimate* (Standard error), p-value	
Group affiliation (ref. control)	−23.537 (4.831), <0.001	−0.403 (0.117), 0.001
Age	−0.568 (0.481), 0.607	−0.004 (0.012), 0.719
Gender (ref. female)	−8.188 (4.912), 0.098	0.070 (0.119), 0.557
Smoking (ref. no)	2.062 (4.912), 0.675	0.283 (0.118), 0.018
BMI	0.310 (0.602), 0.607	−0.005 (0.015), 0.738
FPG at baseline	0.541 (0.053), <0.001	0.795 (0.044), <0.001
HbA1c at baseline	R^2^ = 0.539, p <0.001	R^2^ = 0.757, p <0.001

^*^Adjusted for group affiliation, age, gender, smoking, BMI, and the respective baseline measures.

**Table 5:** Multiple linear regression analysis for periodontal status (plaque index, gingival index, pocket depth, CAL, and BOP) (N = 130)

**Variables**	**Plaque index**	**Gingival index**	**Pocket depth**	**CAL**	**BOP**
	**(3rd month)**	**(3rd month)**	**(3rd month)**	**(3rd month)**	**(3rd month)**
	Parameter estimate* (Standard error), p-value				
Group affiliation (ref. control)	−0.148 (0.062), 0.018	−0.172 (0.058), 0.004	−0.480 (0.091), <0.001	−0.374 (0.111), 0.001	−18.938 (4.406), <0.001
Age	0.008 (0.006), 0.170	−0.002 (0.006), 0.712	0.002 (0.009), 0.826	0.010 (0.011), 0.372	−0.245 (0.434), 0.573
Gender (ref. female)	0.072 (0.064), 0.261	0.008 (0.060), 0.888	0.101 (0.094), 0.284	0.148 (0.117), 0.209	6.366 (4.587), 0.168
Smoking (ref. no)	0.166 (0.064), 0.011	0.011 (0.060), 0.857	−0.091 (0.097), 0.352	0.029 (0.118), 0.808	2.437 (4.549), 0.593
Plaque index at baseline	0.346 (0.075), <0.001				
Gingival index at baseline		0.313 (0.066), <0.001			
Pocket depth at baseline			0.732 (0.068), <0.001		
CAL at baseline				0.836 (0.053), <0.001	
BOP at baseline					0.585 (0.092), <0.001
	R^2^ = 0.238, p <0.001	R^2^ = 0.200, p <0.001	R^2^ = 0.533, p <0.001	R^2^ = 0.721, p <0.001	R^2^ = 0.318, p <0.001

*Adjusted for group affiliation, age, gender, smoking, and the respective baseline measures.

## Discussion

The present study shows that the combination of lifestyle change and dental care in one program, improved both glycemic and periodontal status in the elderly with type 2 diabetes.

As mentioned in the introduction, combined lifestyle change and periodontal care intervention are needed to prevent dental complications. The multiple linear regression analysis showed that the LCDC program was significantly negatively correlated in both glycemic (FPG and HbA1c) and periodontal status (plaque index, gingival index, pocket depth, CAL, and BOP). Furthermore, the multiple linear regression analysis also found HbA1c and the plaque index score significantly correlated with smoking which is consistent with a previous study that found that smoking is a risk factor for both periodontal disease and DM [19,20].

After the LCDC program, glycemic status including FPG and HbA1c decreased with statistically significant differences between the intervention and the control groups at 3 month follow up. Whereas, in the control group, both FPG and HbA1c increased from baseline to 3 month follow up. The results showed an improvement of glycemic status in the intervention group and deterioration of glycemic status in the control group. The difference of HbA1c in the intervention and the control groups were −0.29% and +0.09%, respectively with a statistically significant difference consistent with a previous study regarding the effect of changes in diet on HbA1c for 3 months which found the difference of HbA1c in the intervention was −0.83% with a statistically significant difference [21]. In other previous studies, meal preparation training also decreased HbA1c (0.3%) after 6 month follow up [22] and lifestyle counseling in the primary care setting also decreased HbA1c [23]. However, the effect of changes in supportive telephone counseling on HbA1c for 18 months did not present a statistically significant difference between the intervention and the control groups [24].

After the LCDC program, periodontal status including plaque index, gingival index, pocket depth, CAL, and BOP decreased from baseline to 3 month follow up. The slight decrease in mean differences of periodontal status highlighted the first step of the periodontal disease improvement with statistically significant differences between the intervention and the control groups at 3 month follow up. The present study found the significant correlation between periodontal status (BOP) and glycemic status (FPG) after the LCDC program was consistent with the previous studies that found periodontal disease is associated with the progression of HbA1c [25,26]. Many studies in other countries have found periodontal therapy including tooth brushing instruction, oral health education, and supra-gingival scaling improved glycemic control by decreasing HbA1c and periodontal status with statistically significant differences [9,10]. However, a previous study in Thailand found a decrease of HbA1c after periodontal treatment by scaling and root planing without a statistically significant difference [12].

Previous research papers which studied knowledge and attitude toward oral health and DM in type 2 diabetes have found the scores of knowledge and attitude in diabetic patients were low to moderate [27-30]. The present study found knowledge and attitude toward oral health and DM increased after the intervention. These results showed the effectiveness of the LCDC program to increase and maintain knowledge and attitude of the elderly with type 2 diabetes for 3 months. This is consistent with a previous study which found diabetic patients who received oral health information related to diabetes by health professionals, knowledge scored 2.9 times higher, compared to participants who did not receive that information [31].

The present study found the participants in the intervention group more likely to exercise, modify diet, have foot examinations, always wear covered shoes, and participate in screening of feet than the participants in the control group. This is inconsistent with a previous research paper, which studied a structured group diabetes education program of 6 hours, which was delivered in the community, with follow up for 3 years. The research found no statistically significant difference of physical activity [8]. The difference between the results of the current study and the abovementioned study is due to the fact that the abovementioned study used group education, did not use educational boosters, and used long term follow up. However, the present study used a mix of individual and group education, which was boosted every month and used short term follow up. The results of the present study are consistent with a previous study, which found improvements in eating control and step counts after receiving meal preparation training [22]. Yet another previous study found the association between the knowledge of preventive behaviors regarding foot ulcers and actual preventive behaviors [32] to be consistent with the present study which found the participants in the intervention group increased their knowledge score and improved their foot behaviors after receiving the LCDC program.

The results of the present study show knowledge and attitude toward oral health and DM in the elderly with type 2 diabetes translated to practice in both oral health and DM.

The strengths of the present study are the high response rate (98.5%) and that it used biomarkers including HbA1c, FPG, plaque index score, gingival index score, pocket depth, and CAL to examine the outcomes.

The limitations of the present study are a lack of random assignment due to a quasi-experimental design, selection bias from the willingness to participate and it was not representative of the entire elderly population with type 2 diabetes because of the small number of centers in which the present study was conducted. However, the LCDC program had the effectiveness and acceptability that could be adapted into routine work by staff in the health centers which could be implemented in the other health centers. The single-blind technique might cause measurement bias. Furthermore, the use of participant reports to estimate practice toward DM and oral health behaviors are subject to some degree of measurement error.

Future studies need to incorporate a longer follow-up period to generate understanding of intervention effects, adherence and sustainability, over time, by randomized controlled trial.

## Conclusion

The LCDC program was significantly negatively correlated in both glycemic and periodontal status that improved the glycemic status (HbA1c and FPG) and periodontal status (plaque index score, gingival index score, pocket depth, CAL, and BOP), which were maintained for 3 months. Furthermore, the results of the present study show the effectiveness of the LCDC program by increasing knowledge, attitude and practice toward oral health and DM of the elderly with type 2 diabetes.

## Abbreviations

BOP: Bleeding on probing; DM: Diabetes mellitus; HbA1c: Glycosylated hemoglobin; FPG: Fasting plasma glucose; MI: Motivational interviewing; BMI: Body mass index; CAL: Clinical attachment level; CI: Confidence interval; LCDC program: Lifestyle change plus dental care program.

## Competing interests

The authors declare that they have no competing interests.

## Authors’ contributions

SS participated in the sequence alignment, drafted the manuscript, designed the study and performed the statistical analysis. ST conceived the study, participated in its design and coordination, and helped to draft the manuscript. Both authors read and approved the final manuscript.

## Authors’ information

Saruta Saengtipbovorn is a dentist at Health Center 54 and a doctoral student in Public Health at the College of Public Health Sciences, Chulalongkorn University. Surasak Taneepanichskul is a medical doctor, Professor of Obstetrics and Gynaecology and Dean of the College of Public Health Sciences, Chulalongkorn University.

## Pre-publication history

The pre-publication history for this paper can be accessed here:

http://www.biomedcentral.com/1472-6831/14/72/prepub

## References

[B1] Scully C Ettinger RL The influence of systemic diseases on oral health care in older adults J Am Dent Assoc 2007 138 Suppl 7S 14S 17761840 10.14219/jada.archive.2007.0359

[B2] Albert DA Ward A Allweiss P Graves DT Knowler WC Kunzel C Leibel RL Novak KF Oates TW Papapanou PN Schmidt AM Taylor GW Lamster IB Lalla E Diabetes and oral disease: implications for health professionals Ann N Y Acad Sci 2012 1255 1 15 22409777 10.1111/j.1749-6632.2011.06460.x PMC3429365

[B3] LE Lamster IB Borgnakke WS Taaylor GW The relationship between oral health and diabetes mellitus JAMA 2008 139 suppl 19 24 10.14219/jada.archive.2008.0363 18809650

[B4] Jarvis JST Carey M Davies M How can structured self-management patient education improve outcomes in people with type 2 diabetes? Diabet Obes Metab 2010 12 12 19 10.1111/j.1463-1326.2009.01098.x 19788430

[B5] Vermunt PW Milder IE Wielaard F Baan CA Schelfhout JD Westert GP van Oers HA Implementation of a lifestyle intervention for type 2 diabetes prevention in Dutch primary care: opportunities for intervention delivery BMC Fam Pract 2012 13 79 22873753 10.1186/1471-2296-13-79 PMC3457845

[B6] Noda K Zhang B Iwata A Nishikawa H Ogawa M Nomiyama T Miura S Sako H Matsuo K Yahiro E Yanase T Saku K Lifestyle change through the use of delivered meals and dietary counseling in a Single-blind study Circ J 2012 76 1335 1344 22739083 10.1253/circj.cj-12-0164

[B7] Lorgerol M Commentary on the clinical management of metabolic syndrome: why a healthy lifestyle is important BMC Med 2012 10 139 149 23151252 10.1186/1741-7015-10-139 PMC3520757

[B8] Khunti KGL Skinner T Carey ME Realf K Dallosso H Fisher H Campbell M Heller S Davies MJ Effectiveness of a Diabetes Education and Self Management Programme (DESMOND) for people with newly diagnosed type 2 diabetes mellitus: three year follow-up of a cluster randomized controlled trial in primary care BMJ 2012 344 e2333 22539172 10.1136/bmj.e2333 PMC3339877

[B9] Long O Li RF Effect of periodontal treatment on glycosylated hemoglobin levels in elderly patients with periodontal disease and type 2 diabetes Chin Med J 2011 124 3070 3073 22040558

[B10] Singh SKV Kumar S Subbappa A The effect of periodontal therapy on the improvement of glycemic control in patients with type 2 diabetes mellitus: a randomized controlled clinical trial Int J Diabetes Dev Ctries 2008 28 38 44 19902046 10.4103/0973-3930.43097 PMC2772010

[B11] Sun WLCL Zhang SZ Wu YM Ren YZ Qin GM Inflammatory cytokines, adiponectin, insulin resistance and metabolic control after periodontal intervention in patients with type 2 diabetes and chronic periodontitis Intern Med 2011 50 1569 1574 21804283 10.2169/internalmedicine.50.5166

[B12] Promsudthi APS Deerochanawong C Kanchanavasita W The effect of periodontal therapy on uncontrolled type 2 diabetes mellitus in older subjects Oral Disease 2005 11 293 298 10.1111/j.1601-0825.2005.01119.x 16120115

[B13] Teeuw WJGV Loos BG Effect of periodontal treatment on glycemic control of diabetic patients Diabetes Care 2010 33 421 427 20103557 10.2337/dc09-1378 PMC2809296

[B14] Centers for Disease Control and Prevention (CDC) Controlling your diabetes [Internet] 2011 [Cited 2013, June 27] Available from: http://www.cdc.gov/diabetes/pubs/tcyd/control.htm

[B15] U.S. Department of Health and Human Services Theory at a Glance: A Guide for Health Promotion Practice 2005 Washington: U.S. Department of Health and Human Services

[B16] Codental Indices Used for Periodontal Disease Assessment [Internet] 2012 [Cited 2013, June 30] Available from: http://www.codental.uobaghdad.edu.iq/uploads/lectures/3rd%20class%20community%20dentistry/3%20PDD%20Indices.pdf

[B17] Eickholz P Clinical periiodontal diagnosis: probing pocket depth, vertical attachment level and bleeding on probing Perio 2004 1 75 80

[B18] Division of Periodontology, University of Minnesota Advanced Probing Techniques [Internet] 2013 [Cited 2013, June 30] Available from: http://www1.umn.edu/perio/dent5612-04/module_21.pdf

[B19] Bergman BC Perreault L Hunerdosse D Kerege A Playdon M Samek AM Eckel RH Novel and reversible mechanisms of smoking-induced insulin resistance in humans Diabetes 2012 61 12 3156 3166 22966072 10.2337/db12-0418 PMC3501865

[B20] National Institute of Dental and Craniofacial Research (NIDCR) Periodontal (Gum) disease: causes, symptoms, and treatments [Internet] 2013 Available from: http://www.nidcr.nih.gov/OralHealth/Topics/GumDiseases/PeriodontalGumDisease.htm

[B21] Miller CKKJ Heading A Nagaraja A Miser WF Comparative effectiveness of a mindful eating intervention to a diabetes self-management intervention among adults with type 2 diabetes: a pilot study J Acad Nutr Diet 2012 112 1835 1842 23102183 10.1016/j.jand.2012.07.036 PMC3485681

[B22] Dasgupta K Hajna S Joseph L Da Costa D Christopoulos S Gougeon R Effects of meal preparation training on body weight, glycemia, and blood pressure: results of a phase 2 trial in type 2 diabetes Int J Behav Nutr Phys Act 2012 9 125 23075398 10.1186/1479-5868-9-125 PMC3543247

[B23] Morrison FSM Turchin A Lifestyle counseling in routine care and long-term glucose, blood pressure and cholesterol control in patients with diabates Diabetes Care 2012 35 334 341 22275442 10.2337/dc11-1635 PMC3263885

[B24] Mons U Raum E Kramer HU Ruter G Rothenbacher D Rosemann T Szecsenyi J Brenner H Effectiveness of a supportive telephone counseling intervention in type 2 diabetes patients: randomized controlled study PLoS ONE 2013 8 10 e77954 24205043 10.1371/journal.pone.0077954 PMC3813502

[B25] Demmer RT Desvarieux M Holtfreter B Jacobs DR Jr Wallaschofski H Nauck M Volzke H Kocher T Periodontal status and A1C change: longitudinal results from the study of health in Pomerania (SHIP) Diabetes Care 2010 33 5 1037 1043 20185742 10.2337/dc09-1778 PMC2858171

[B26] Kim EK Lee SG Choi YH Won KC Moon JS Merchant AT Lee HK Association between diabetes-related factors and clinical periodontal parameters in type-2 diabetes mellitus BMC Oral Health 2013 13 64 24195646 10.1186/1472-6831-13-64 PMC3829373

[B27] Al-Maskari F El-Sadig M Al-Kaabi JM Afandi B Nagelkerke N Yeatts KB Knowledge, attitude and practices of diabetic patients in the United Arab Emirates PLoS ONE 2013 8 e52857 23341913 10.1371/journal.pone.0052857 PMC3544806

[B28] Upadhyay DKPS Shanker PR Mishra P Knowledge, attitude and practice about diabetes among diabetes patients in Western Nepal Rawal Med J 2008 33 8 11

[B29] Ismaeil FMAN Diabetic patients knowledge, attitude and practice toward oral health JEP 2013 4 19 25

[B30] Eknithiser RHP Havanond P Knowledge, attitude, and practice (KAP) of diabetes mellitus type II patients in multidisciplinary program at diabetes mellitus clinic, Phanomphrai Hospital, Phanomphrai District, Roi-et Province, Thailand J Health Res 2010 24 suppl 2 87 92

[B31] Yuen HKWB Bandyopadhyay D Magruder KM Salinas CF London SD Oral health knowledge and behavior among adults with diabetes Diabetes Res Clin Pract 2009 86 239 246 19800143 10.1016/j.diabres.2009.09.010 PMC2791496

[B32] Kim AYHP Preventive behaviors regarding foot ulcers in diabetes type II patients at BMA health center no.48, Bangkok, Thailand J Health Res 2008 22 suppl 21 28

